# Cancer Cell B7-H3 Expression Is More Prevalent in the Pancreato-Biliary Subtype of Ampullary Cancer Than in Pancreatic Cancer

**DOI:** 10.3389/fonc.2021.615691

**Published:** 2021-04-29

**Authors:** Emma E. Geerdes, Kostandinos Sideras, M. Hosein Aziz, Casper H. van Eijck, Marco J. Bruno, Dave Sprengers, Patrick P. C. Boor, Jaap Kwekkeboom

**Affiliations:** ^1^ Department of Gastroenterology and Hepatology, Erasmus MC-University Medical Center, Rotterdam, Netherlands; ^2^ Department of Surgery, Erasmus MC-University Medical Center, Rotterdam, Netherlands

**Keywords:** cancer immunotherapy, CD276, B7H3, immune checkpoint, pancreas

## Abstract

B7-H3 is an immunomodulatory member of the B7-superfamily with limited expression in normal tissues, but overexpression in several types of cancer. Therefore it is currently being explored as a potential target for cancer immunotherapy. The biological relevance of B7-H3 expression in pancreatic cancer is unclear, while there are no data on B7-H3 expression in ampullary cancer. We aimed to compare intra-tumoral B7-H3 expression between these two closely related cancer types and analyze its association with post-surgical disease course. B7-H3 expression levels were determined by immunohistochemistry in tissue microarrays of resected tumors of 137 pancreatic cancer patients and 83 patients with ampullary cancer of the pancreato-biliary subtype. B7-H3 was more frequently expressed in cancer cells of ampullary cancer patients compared to pancreatic cancer patients (51% *versus* 21%; p< 0.001). In ampullary cancer patients, but not in pancreatic cancer patients, B7-H3 cancer cell expression was associated with longer disease-free survival and patient survival. However, the prognostic value of B7-H3 was lost upon adjustment for CA19-9 levels. The frequencies of B7-H3 expression in tumor stroma did not differ between the two types of cancer (66% versus 63%). In both cancer types, stromal B7-H3 expression was not associated with post-surgical disease course. Compared to pancreatic cancer, B7-H3 is more frequently expressed in cancer cells of patients with the pancreato-biliary subtype of ampullary cancer. These data suggest that B7-H3 may represent an interesting potential target for immunotherapy in ampullary cancer rather than in pancreatic cancer.

## Introduction

Pancreatic carcinoma is a highly fatal form of cancer (1). Due to its asymptomatic early stage, 80 to 85% of patients present with late, non-resectable disease and have a dismal prognosis (2). While newer chemotherapeutic treatments have somewhat improved the median survival of patients with pancreatic adenocarcinoma, both in the locally advanced (3), and metastatic setting (4, 5), overall prognosis remains dismal. Moreover, pancreatic adenocarcinoma is one of the few malignancies whose age-adjusted population mortality is increasing (https://www.cancer.org/research/cancer-facts-statistics.html) (2). Patients who are eligible for surgical resection have a longer, but still limited, life expectancy compared to other cancers, with a 5-year survival rate of 39% (https://www.cancer.org/research/cancer-facts-statistics.html) and that is despite improvements in modern adjuvant chemotherapeutic treatments for early pancreatic cancer (6). Ampullary cancer is a more uncommon cancer type, which originates from the Ampulla of Vater (7). The resectability rate of ampullary cancer is much higher (88%) (8), but the majority of patients eventually die due to disease recurrence. Two different histological subtypes of ampullary adenocarcinomas, intestinal and pancreato-biliary, are distinguished based on their epithelium of origin (9). The pancreato-biliary subtype often grows into the pancreas, is histologically similar to pancreatic cancer, and has the worst prognosis (5 year survival rate of 20%) (10, 11). Ampullary cancers are highly resistant to current chemotherapeutic and radio-therapeutic treatments (12). Therefore, there is an urgent need for more effective treatment strategies for both types of cancer.

Immunotherapy using antibodies that block interactions between inhibitory immune checkpoint receptors on T cells and their ligands on tumor cells and/or immune cells, such as binding of CTLA-4 to B7-1 and/or B7-2, and binding of PD1 to PD-L1, thereby unleashing inhibition of anti-tumor T-cells, has shown considerable clinical benefits in several types of solid cancers (13). Whereas immune checkpoint antibody therapy has not yet been studied in ampullary cancer, anti-PD-L1 antibodies and anti-CTLA-4 antibodies have been found to be clinically ineffective in pancreatic cancer (14, 15). Therefore, it is important to identify other molecular targets for the development of immunotherapeutic strategies against this type of cancer.

B7-H3 (CD276) is a member of the B7 superfamily member that is expressed by monocytes and dendritic cells. When initially discovered, B7-H3 was thought to be co-stimulatory for T cells (16), but later studies showed that B7-H3 generally serves as a co-inhibitory immune checkpoint for T-cells (17–20). The receptor of B7-H3 is still unknown (21, 22). Despite broad mRNA expression, B7-H3 has limited protein expression in healthy human tissues, suggesting tight post-transcriptional regulation. In contrast, B7-H3 is overexpressed by cancer cells of many solid cancer types (22), probably due to down-regulation of microRNA-29 in tumors (23). B7-H3 is considered as an attractive target for cancer immunotherapy due to its broad overexpression across multiple tumor types and low expression in healthy tissues. In several mouse tumor models, including pancreatic cancer (24, 25), antibody blockade of B7-H3 inhibits tumor growth and enhances tumor-specific CD8+ T-cell responses and CD8+ T-cell infiltration into tumors (26, 27). In addition to blockade of its T-cell co-inhibitory function, B7-H3 has also potential as a tumor-specific target for direct induction of tumor cell death. An Fc-optimized anti-B7-H3 antibody exhibited potent anti-tumor activity in mouse tumor models through antibody-dependent cell-mediated cytotoxicity (ADCC) (28). This antibody is currently being evaluated in patients with diverse types of solid cancers (29). In addition, an anti-B7-H3 antibody-cytotoxic drug conjugate (30) and chimeric antigen receptor (CAR)-transfected T cells targeting B7-H3 (31) showed anti-tumor activity in different experimental animal tumor models without evident toxicity. More recently, B7-H3 was found to be overexpressed on tumor-associated vasculature and stromal fibroblasts in several cancer types, including pancreatic cancer (30–33), which suggests that B7-H3-directed antibodies or CAR-T cells may be able to target not only cancer cells, but also tumor stroma and vasculature, both of which can have tumor-promoting functions (34, 35).

B7-H3 expression has been studied in many solid cancer types, including pancreatic cancer (24, 32, 36–42), but not in ampullary cancer. Moreover, whether and how B7-H3 expression in pancreatic cancer cells is associated with prognosis is a matter of controversy. One study concluded that tumoral B7-H3 expression in pancreatic cancer patients is associated with improved patient survival (36), whereas other studies found that it is associated with a worse survival (32, 39, 40, 42) or found that there is no association with patient survival (41). Moreover, only few studies have investigated B7−H3 expression in tumor stroma in pancreatic cancer separately, and none of them analyzed whether stromal B7-H3 expression is associated with prognosis (30–32).

Therefore, the aims of this study are to compare the frequencies of B7-H3 protein expression in cancer cells and tumor stroma of pancreatic and ampullary cancer patients, and to study their association with baseline clinicopathologic factors and patient outcome. For ampullary cancer, we focused on the pancreato-biliary subtype because of its similarities with pancreatic cancer.

## Patients and Methods

### Patient Population and Tissue Samples

This study was performed using tissue micro-arrays (TMA) containing five 1-mm cores from formalin fixed paraffin-embedded tumor tissues of 220 patients who underwent resection of pancreatic cancer (n=137) or pancreato-biliary subtype of ampullary cancer (n=83) at the Erasmus University Medical Center (Erasmus MC) between December 2000 and December 2018. Further details on TMA construction can be found in a previous paper (43). Baseline clinicopathologic characteristics were retrospectively collected from electronic patient records. All patients were treatment-naïve before tumor resection. Follow-up information was updated until December 31, 2018. Patients who died from postoperative complications were excluded from survival analysis. The median follow-up duration of pancreatic cancer patients was 16 months (range: 0.2 – 156) and of ampullary cancer patients 21 months (range 0.1-177). The study protocol was approved by the Medical Ethical Committee of Erasmus MC. Clinicopathologic information of the patients is shown in **Table 1**.

**Table 1:** Cancer cell expression of B7-H3 and baseline characteristics of pancreatic cancer and ampullary cancer patients.

Total (n=220)	Pancreas cancer (n=137)			Ampullary cancer (n=83)		
Tumor cell expression:	B7-H3 Negative(n=108)	B7-H3 Positive(n=29)	P Value	B7-H3 Negative(n=41)	B7-H3 Positive(n=42)	P Value^5^ 0.000
**Age in years^1^**	69.3 (33.4 – 81.4)	65.0 (35.5 – 79.2)	0.116	67.5 (43.8 – 80.0)	69.6 (42.2 – 85.2)	0.483
male (n=132)	58 (76.3%)	18 (23.7%)	0.529	30 (53.6%)	26 (46.4%)	0.350
Female (n=88)	50 (82.0%)	11 (18%)		11 (40.7%)	16 (59.3%)	
**Positive margins^2^**						
No (n=140)	52 (76.5%)	16 (23.5%)	0.676	33 (45.8%)	39 (54.2%)	0.116
Yes (n=79)	55 (80.9%)	13 (19.1%)		8 (72.7%)	3 (27.3%)	
**Lymph nodes metastasis**						
No (n=81)	32 (71.1%)	13 (28.9%)	0.181	16 (44.4%)	20 (55.6%)	0.509
Yes (n=139)	76 (82.6%)	16 (17.4%)		25 (53.2%)	22 (46.8%)	
**CA-19.9 (kU/l)^1,3^**	94.0 (1 - 6556)	61.0 (1 – 998)	0.318	77 (1 – 2242)	38 (1 – 718)	0.052
**Differentiation**						
Good (n=18)	7 (77.8%)	2 (22.2%)	1.000	4 (44.4%)	5 (55.6%)	0.764
Moderate (n=131)	65 (79.3%)	17 (20.7%)		23 (46.9%)	26 (53.1%)	
Poor (n=71)	36 (78.3%)	10 (21.7%)		14 (56.0%)	11 (44.0%)	
**T-stage (8^th^ edition AJCC/UICC 2016)**						
T1 (n=40)	21 (91.3%)	2 (8.7%)	0.222	5 (29.4%)	12 (70.6%)	0.296
T2 (n=118)	72 (74.2%)	25 (25.8%)		12 (57.1%)	9 (42.9%)	
T3 (n=58)	14 (87.5%)	2 (12.5%)		22 (52.4%)	20 (47.6%)	
T4 (n=4)	1 (100%)	0 (0%)		2 (66.7%)	1 (33.3%)	
**Any post-operative chemotherapy**						
No (n=120)	47 (78.3%)	13 (21.7%)	1.000	28 (46.7%)	32 (53.3%)	0.469
Yes (n=100)	61 (79.2%)	16 (20.8%)		13 (56.5%)	10 (43.5%)	
**Adjuvant therapy^4^**						
No (n=71)	56 (78.9%)	15 (21.1%)	1.000	32 (47.8%)	35 (52.2%)	0.569
Yes (n=62)	49 (79.0%)	13 (21.0%)		9 (60.0%)	6 (40.0%)	

^1^Median (range).

^2^Margin status of 1 pancreas cancer patient is unknown.

^3^Last pre-surgical value.

^4^Adjuvant therapy of 4 pancreas cancer patients and of 1 ampullary cancer patient are unknown.

^5^After Bonferroni correction, a p-value < 0.05/9=0.006 is considered as significant.

Age and CA-19.9 Mann Whitney U-test, others Fischer’s exact test (2-sided).

### B7H3 Immunohistochemistry

B7-H3 was immunohistochemically stained using a specific rabbit monoclonal antibody (clone SP206) obtained from Sigma-Aldrich Chemie N.V., Zwijndrecht, Netherlands, which has been used in two previous studies (33, 44). TMA sections were deparaffinized followed by antigen retrieval in 10 mM sodium citrate buffer (pH 6.0) in a microwave for 10 minutes. Endogenous peroxidase activity was blocked in 0.3% H_2_O_2_ for 15 minutes. After using goat serum for blocking, primary anti-B7-H3 antibody was applied at 4°C overnight. HRP-conjugated goat anti-mouse IgG polymer secondary antibody (EnvisionTM, DAKO) was then applied for 1 hour at room temperature, followed by DAB as chromogen. The slides were counter-stained with hematoxylin. Omission of the primary antibody served as negative control stains, while TMA’s containing healthy human tissue cores were used to validate the specificity and determine the optimal dilution of the primary antibody. The slides were scanned using a Hamamatsu NanoZoomer 2.0HT, and visualized by NDP-viewer version 2 software. Staining intensity of B7-H3 in cancer cells and in tumor stroma was scored separately, for each core, as absent, low, intermediate or strong by two independent investigators (EG and PPCB) blinded to clinical outcome. Differences between individual scores were resolved by mutual agreement. In 11% of patients B7-H3 expression levels in cancer cells showed differences between individual tumor tissue cores, and in 28% of patients B7-H3 expression levels in stromal cells varied between individual tumor tissue cores, reflecting heterogeneity in expression levels between different parts of the tumors. In these cases, median scores from the 5 tumor cores were used for further analysis.

### Statistical Analysis

Differences between tumor types and associations between clinic-pathologic characteristics and B7-H3 expression were examined using the Fischer’s exact test or the Kruskal-Wallis test as appropriate. Survival curves were estimated by the Kaplan-Meier method, and the log-rank test was used to evaluate differences between survival curves of different groups. Cancer-specific survival and recurrence-free survival were calculated from the date of surgery to the date of event (death from cancer or recurrence of cancer, respectively). The Cox proportional hazard regression analysis was used for multivariable analysis. The statistical analysis was performed using the SPSS^©^ 25 software.

## Results

### B7-H3 Expression in Cancer Cells and Tumor Stroma of Pancreatic and Ampullary Cancer

**Figure 1:**
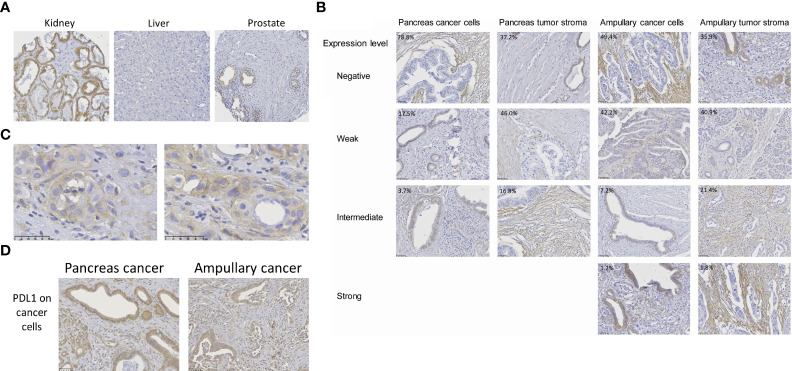
B7-H3 expression in pancreas cancer, ampullary cancer and healthy tissues. **(A)** B7-H3 expression in human kidney, prostate and liver, as determined by immunohistochemistry using the SP206 monoclonal antibody. For kidney, the tissue with the highest expression in our TMA is shown. **(B)** B7-H3 expression levels in cancer cells and in tumor stroma were scored separately, as negative, weak, intermediate, or strong. B7-H3 expression in cancer cells was predominantly visible in the cytoplasm, but in some cases (e.g. in the pancreatic cancer with intermediate level of B7-H3 expression in cancer cells) additional membrane expression was observed. In stromal cells, B7-H3 expression was more often localized at the cell membrane. There were no pancreatic tumors with high B7-H3 expression in cancer cells or in stroma. Percentages depicted in the pictures are percentages of tumors with the level of expression shown in the respective pictures. **(C)** Two additional examples of distinctive B7-H3 membrane expression in cancer cells of pancreatic cancer patients. **(D)** For comparison, examples of PD-L1 expression in cancer cells of both cancer types is shown.

Specificity of B7-H3 immunohistochemistry was established by evaluation of the staining of healthy human tissue cores in TMA’s. In accordance with published B7-H3 immunohistochemistry data, we observed variable membrane and cytoplasmic B7-H3 expression in tubular epithelium of kidney (45), no or at most very low expression in healthy liver (30), and weak expression (mainly membranous) in epithelial cells of prostate (30, 31, 46) (**Figure 1A**).

In agreement with previous studies (24, 31, 36–38, 40, 41), B7-H3 expression in cancer cells of pancreatic and ampullary cancer patients was predominantly visible in the cytoplasm. In some cases additional membrane expression was observed, but in most tumors membrane expression was difficult to distinguish from cytoplasmic expression (**Figures 1B, C**). We never saw membrane expression without cytoplasmic expression in cancer cells. In stromal cells, B7-H3 expression was more often localized at the cell membrane. To prevent under-estimation of B7-H3 expression, we assessed B7-H3 expression in as absent, weak, intermediate and strong without distinguishing membrane and cytoplasmic expression (**Figure 1B**). In 29 of 137 (21%) pancreatic cancer patients we observed weak (17.5%) to intermediate (3.7%) B7-H3 expression in cancer cells, whereas in 63% of these patients weak (46%) to intermediate (17%) B7-H3 expression in tumor stroma was found. Strong B7-H3 expression was not observed in pancreatic cancer. Thus, a minority of pancreatic cancers have weak to intermediate B7-H3 expression in cancer cells, whereas a majority show weak to intermediate expression of B7-H3 in tumoral stroma.

Cancer cell B7-H3 expression was more frequently observed in ampullary cancer patients (42 of 83 patients (51%), versus 21% of pancreatic cancer patients; p< 0,001), with 1 of these patients even showing strong B7-H3 expression in tumor cells. However, frequencies of patients with B7-H3 expression in tumor stroma did not differ between the two types of cancer (66% of ampullary tumors versus 63% of pancreatic tumors; p=0.664). Nevertheless, compared to pancreatic tumors, more ampullary tumors showed intermediate to strong stromal B7-H3 expression (23% of ampullary tumors versus 17% of pancreatic tumors; p= 0.04).

The frequency of B7-H3 expression is much lower than that of PD-L1 expression in cancer cells of these patients. Previously, we reported that 90% of patients of this cohort express at least some level of PD-L1 in their cancer cells (43). Similar to B7-H3, PD-L1 expression was mainly found in the cytoplasm of cancer cells (**Figure 1D**).

### Associations Between B7-H3 Expression and Baseline Clinicopathologic Characteristics and Post-Surgical Outcome

**Figure 2:**
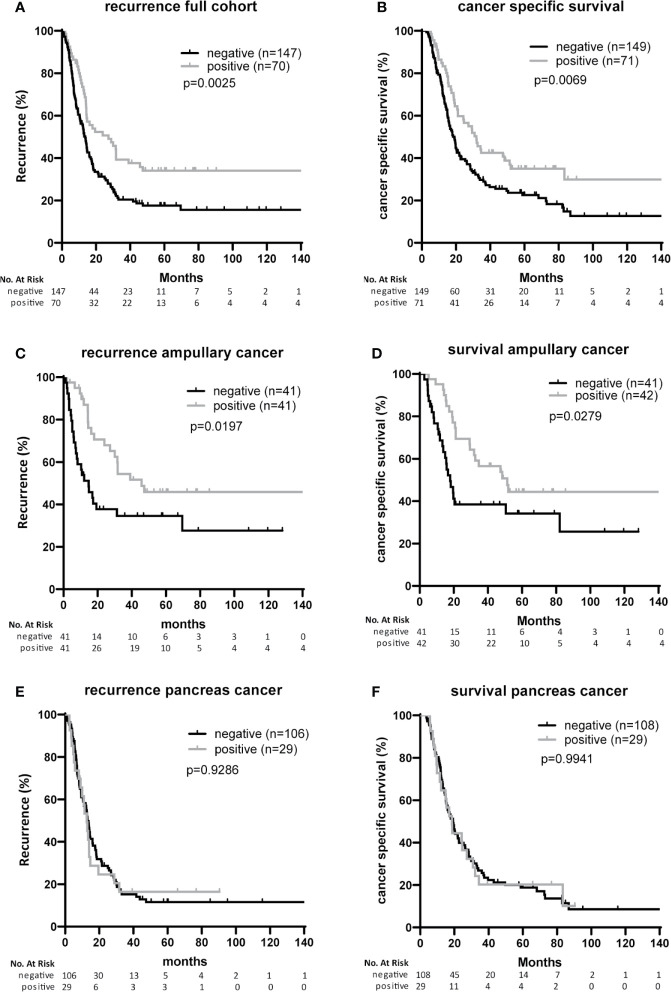
Associations of B7-H3 expression on cancer cells with patient survival and tumor recurrence **(A, B)**. Full cohort. Kaplan Meier plots showing recurrence-free survival time **(A)** and cancer-specific patient survival **(B)** after resection of tumors with or without B7-H3 expression in cancer cells. **(C, D)** Ampullary cancer patients. Kaplan Meier plots showing recurrence-free survival time **(C)** and cancer-specific patient survival **(D)** of patients after resection of tumors with or without B7-H3 expression in cancer cells. **(E, F)** Pancreatic cancer patients. Kaplan Meier plots showing recurrence-free survival time **(E)** and cancer-specific patient survival **(F)** of patients after resection of tumors with or without B7-H3 expression in cancer cells.

After Bonferroni correction for testing of 9 variables, no significant associations were observed between B7-H3 expression in cancer cells or tumor stroma and baseline clinicopathologic characteristics (all p-values > p=0.05/9 = 0.006; **Tables 1** and **2**). In the full cohort, expression of B7-H3 in cancer cells was significantly associated with less cancer recurrence and improved post-operative cancer-specific patient survival (**Figures 2A, B**). When analyzing the two cancer types separately, cancer cell B7-H3 expression was positively associated with recurrence and survival in ampullary cancer patients (**Figures 2C, D**), but not in pancreatic cancer patients (**Figures 2E, F**). No significant associations were observed between stromal B7-H3 expression and post-surgical disease course (data not shown).

**Table 2:** Tumor stroma expression of B7-H3 and baseline characteristics of pancreatic cancer and ampullary cancer patients.

Total (n=220)	Pancreas cancer (n=137)			Ampullary cancer (n=83)		
Tumor stroma expression	B7H3 Negative (n=51)	B7H3 Positive(n=86)	P Value	B7H3 Negative (n=28)	B7H3 Positive (n=55)	P Value^5^ 0.664
**Age in years^1^**	65.7 (40.0 – 79.2)	69.0 (33.4 – 81.4)	0.289	67.5 (42.2 – 83.9)	68.2 (43.8 – 85.2)	0.931
**Gender**						
male (n=132)	25 (32.9%)	51 (67.1%)	0.287	21 (37.5%)	35 (62.5%)	0.333
Female (n=88)	26 (42.6%)	35 (57.4%)		7 (25.9%)	20 (74.1%)	
**Positive margins^2^**						
No (n=140)	26 (38.2%)	42 (67.8%)	1.000	21 (29.2%)	51 (70.8%)	0.038
Yes (n=79)	25 (36.8%)	43 (63.2%)		7 (63.6%)	4 (36.4%)	
**Lymph nodes metastasis**						
No (n=81)	19 (42.2%)	26 (57.8%)	0.453	15 (41.7%)	21 (58.3%)	0.242
Yes (n=139)	32 (34.8%)	60 (65.2%)		13 (27.7%)	34 (72.3%)	
**CA-19.9 (kU/l)^1,3^**	76.5 (1 – 2699)	86.0 (1-6556)	0.207	44.0 (1 -2242)	47.0 (1-737)	0.836
**Differentiation**						
Good (n=18)	3 (33.3%)	6 (66.7%)	0.280	7 (77.8%)	2 (22.2%)	0.013
Moderate (n=131)	35 (42.7%)	47 (57.3%)		15 (30.6%)	34 (69.4%)	
Poor (n=71)	13 (28.3%)	33 (71.7%)		6 (24.0%)	19 (76.0%)	
**T-stage (8^th^ edition AJCC/UICC 2016)**						
T1 (n=40)	11 (47.8%)	12 (52.2%)	0.119	8 (47.1%)	9 (52.9%)	0.257
T2 (n=118)	31 (32.0%)	66 (68.0%)		5 (23.8%)	16 (76.2%)	
T3 (n=58)	9 (56.3%)	7 (43.8%)		13 (31.0%)	29 (69.0%)	
T4 (n=4)	0 (0%)	1 (100%)		2 (66.7%)	1 (33.3%)	
**Any post-operative chemotherapy**						
No (n=120)	26 (43.3%)	34 (56.7%)	0.215	20 (33.3%)	40 (66.7%)	1.000
Yes (n=100)	25 (32.5%)	52 (67.5%)		8 (34.8%)	15 (65.2)	
**Adjuvant therapy^4^**						
No (n=71)	28 (39.4%)	43 (60.6%)	0.590	22 (32.8%)	45 (67.2%)	1.000
Yes (n=62)	21 (33.9%)	41 (66.1%)		5 (33.3%)	10 (66.7%)	

^1^Median (range).

^2^Margin status of 1 pancreas cancer patient is unknown.

^3^ Last pre-surgical value.

^4^Adjuvant therapy of 4 pancreas cancer patients and of 1 ampullary cancer patient are unknown.

^5^After Bonferroni correction, a p-value < 0.05/9=0.006 is considered as significant.

Age and CA-19.9 Mann Whitney U-test, others Fischer’s exact test (2-sided).

In univariable analysis, B7-H3 expression in cancer cells and moderate-versus-poor tumor differentiation were significantly associated with better post-surgical ampullary cancer patient survival as well as disease-free survival (Hazard ratio’s <1), while higher T-stage, presence of lymph node metastases, and CA-19-9 levels above median values were associated with worse post-surgical outcomes (Hazard ratio’s >1) (**Tables 3A**, **4A**). In multivariable analysis moderate-versus-poor tumor differentiation, presence of lymph node metastases, and high CA19-9 levels remained independent predictors of patient survival and disease-free survival, but B7-H3 cancer cell expression did not (**Tables 3A** and **4A**). We did a second set of regression analyses in which the association between B7-H3 cancer cell expression and disease course was analyzed together with each of the independent prognostic clinicopathologic characteristics separately (**Tables 3B** and **4B**). These analyses revealed that the observed association between B7-H3 cancer cell expression and better post-surgical outcomes remained significant when adjusted for the tumor characteristics differentiation grade and lymph node metastases. In contrast, the association between B7-H3 cancer cell expression and postsurgical outcome disappeared when adjusted for serum biomarker CA19-9. Indeed, patients with B7-H3 expression in cancer cells tended to have lower CA19-9 levels than patients without, although not statistically significant (**Table 1**). This suggests that tumor cells that express B7-H3 secrete less CA19-9. Together, these data show that B7-H3 expression by cancer cells in ampullary cancer patients is associated with better post-surgical disease course independent of tumor characteristics, but should not be used as a prognostic biomarker because CA19-9 levels have a better prognostic value.

Table 3Cox proportional hazard regression analysis of cancer-specific patient survival in ampullary cancer patients.

**Table 3A:** Cancer cell B7-H3 expression and clinico-pathological variables.

Variables	Univariable Analysis			Multivariable analysis		
	*P*	HR	(95% CI)	*P*	HR	(95% CI)
B7H3-expression in cancer cells	**0.031**	**0.525**	**0.293 – 0.942**	0.368	0.747	0.395 – 1.410
T-stage (T2, T3, T4 vs T1)^1^	**0.033**	**2.787**	**1.087 – 7.145**	0.694	0.811	0.286 – 2.300
Tumor differentiation						
Moderate vs poor	**0.000**	**0.269**	**0.141 – 0.512**	**0.000**	**0.263**	**0.128 – 0.540**
Well-differentiated vs poor	0.258	0.582	0.288 – 1.486	0.437	0.668	0.242 – 1.848
Lymph node metastases (yes/no)	**0.001**	**3.149**	**1.625 – 6.105**	**0.000**	**3.696**	**1.780 – 7.671**
CA19-9 (>45 vs <45 kU/L)^2^	**0.016**	**2.084**	**1.145 – 3.792**	**0.028**	**2.052**	**1.083 – 3.890**
Positive margins	0.100	1.905	0.884 – 4.104			
Any post-operative chemotherapy	0.271	1.401	0.768 – 2.556			
Adjuvant therapy	0.988	0.994	0.492 – 2.010			
Age, yrs	0.805	0.996	0.968 – 1.026			

^1^8^th^ edition.

^2^45 kU/L is median value. Bold means that those values are statistically significant.

**Table 3B:** Cancer cell B7-H3 expression and tumor differentiation or Lymph node metastasis or CA19-9.

Variables	Univariable Analysis			Multivariable analysis		
	*P*	HR	(95% CI)	*P*	HR	(95% CI)
B7H3-expression in cancer cells	**0.031**	**0.525**	**0.293 – 0.942**	**0.029**	**0.505**	**0.273 – 0.931**
Tumor differentiation						
Moderate vs poor	**0.000**	**0.269**	**0.141 – 0.512**	**0.000**	**0.279**	**0.146 – 0.533**
B7H3-expression in cancer cells	**0.031**	**0.525**	**0.293 – 0.942**	**0.015**	**0.479**	**0.266 – 0.865**
Lymph node metastasis (yes/no)	**0.001**	**3.149**	**1.625 – 6.105**	**0.000**	**3.349**	**1.720 – 6.520**
B7H3-expression in cancer cells	**0.031**	**0.525**	**0.293 – 0.942**	0.196	0.675	0.372 – 1.225
CA19-9 (>45 vs <45 kU/L)	**0.016**	**2.084**	**1.145 – 3.792**	**0.019**	**2.054**	**1.127 – 3.744**

Bold means that those values are statistically significant.

Table 4Cox proportional hazard regression analysis of time to cancer recurrence in ampullary cancer patients.

**Table 4A:** Cancer cell B7-H3 expression and clinico-pathological variables.

Variables	Univariable Analysis			Multivariable analysis		
	*P*	HR	(95% CI)	*P*	HR	(95% CI)
B7H3-expression in cancer cells	**0.022**	**0.505**	**0.281 – 0.907**	0.418	0.767	0.404 – 1.457
T-stage (T2, T3, T4 vs T1)^1^	**0.035**	**2.739**	**1.076 – 6.970**	0.603	0.757	0.265 – 2.164
Tumor differentiation						
Moderate vs poor	**0.000**	**0.261**	**0.134 – 0.507**	**0.000**	**0.230**	**0.110 – 0.480**
Well-differentiated vs poor	0.242	0.568	0.220 – 1.466	0.351	0.616	0.222 – 1.708
Lymph node metastases (yes/no)	**0.001**	**3.117**	**1.607 – 6.045**	**0.001**	**3.700**	**1.770 – 7.734**
CA19-9 (>45 vs <45 kU/L)^2^	**0.047**	**1.850**	**1.009 – 3.390**	**0.013**	**2.293**	**1.190 – 4.420**
Positive margins	0.091	1.936	0.900 – 4.165			
Any post-operative chemotherapy	0.111	1.621	0.895 – 2.936			
Adjuvant therapy	0.997	0.999	0.494 – 2.019			
Age, yrs	0.837	0.997	0.967 – 1.027			

^1^8^th^ edition.

^2^45 kU/L is median value. Bold means that those values are statistically significant.

**Table 4B:** Cancer cell B7-H3 expression and tumor differentiation or Lymph node metastasis or CA19-9.

Variables	Univariable Analysis			Multivariable analysis		
	*P*	HR	(95% CI)	*P*	HR	(95% CI)
B7H3-expression in cancer cells	**0.022**	**0.505**	**0.281 – 0.907**	**0.025**	**0.488**	**0.261 – 0.913**
Tumor differentiation						
Moderate vs poor	**0.000**	**0.261**	**0.134 – 0.507**	**0.000**	**0.278**	**0.142 – 0.544**
B7H3-expression in cancer cells	**0.022**	**0.505**	**0.281 – 0.907**	**0.010**	**0.459**	**0.254 – 0.832**
Lymph node metastasis (yes/no)	**0.001**	**3.117**	**1.607 – 6.045**	**0.000**	**3.335**	**1.709 – 6.508**
B7H3-expression in cancer cells	**0.022**	**0.505**	**0.281 – 0.907**	0.181	0.661	0.361 – 1.212
CA19-9 (>45 vs <45 kU/L)	**0.047**	**1.850**	**1.009 – 3.390**	**0.050**	**1.835**	**1.000 – 3.369**

Bold means that those values are statistically significant.

## Discussion

This study is the first to show that 50% of patients with the pancreatico-biliary subtype of ampullary cancer express B7-H3 in their cancer cells, whereas two-thirds of these patients express B7-H3 in their tumoral stroma. Interestingly, cancer cell expression, but not stromal expression, of B7-H3 in these patients was associated with delayed cancer recurrence and improved patient survival. However, this association was lost upon adjustment for CA19-9 serum levels, probably because there was a trend of lower CA19-9 levels in patients with B7-H3 cancer cell expression. Cancer cell expression of B7-H3 was observed in only 20% of pancreatic cancer patients, and was not associated with post-surgical disease course in this cancer type. The frequency of B7-H3 expression in tumor stroma was similar to ampullary cancers, but stromal expression levels were lower.

Whereas in stromal cells B7-H3 expression was often predominantly localized at the cell membrane, in cancer cells B7-H3 was mainly visible in the cytoplasm. In many tumors it was impossible to distinguish membrane expression from cytoplasmic expression. The phenomenon of dominant cytoplasmic B7-H3 expression in cancer cells has not only been observed in pancreatic cancer (as mentioned in the Results section), but many cancer types, such as non-small cell lung cancer (25, 47), colorectal cancer (48) and central nervous system tumors (44). It has been concluded that B7-H3 is expressed both on the membrane and in the cytoplasm of cancer cells (22, 49). Therefore, we did not distinguish between membrane expression and cytoplasmic expression in our assessment of B7-H3 expression. Similarly, we have not discriminated between membrane and cytoplasmic expression of PD-L1, Galectin-9, HVEM and HHLA2 in our previous studies in the same patient cohort (43, 50). Whether cytoplasmic B7-H3 staining reflects the presence of alternative splice variants that lack the transmembrane region but are functionally intact, as has been demonstrated for cytoplasmic PD-L1 (51), is currently unknown.

Previous studies reported large differences in frequencies of B7-H3 expression in tumors of pancreatic cancer patients, ranging from 41% to 94% of all patients. We observed a lower frequency of patients with cancer cell B7-H3 expression. Several differences between individual studies may contribute to the reported variation in proportions of pancreatic patients that express B7-H3 in their tumors. Firstly, differences in ethnicity may play a role. While we studied a Caucasian patient cohort, whereas most previous studies investigated Asian patient cohorts (24, 32, 37–42). The lowest previously reported frequency of B7-H3 expressing cancer cells in pancreatic cancer patients (41%) was observed in a patient cohort from the USA, which probably also consisted largely of Caucasian patients (30). Secondly, whereas we carefully discriminated expression in cancer cells from stromal expression, it is unclear whether expression in these two cell types was distinguished in several previous studies (37, 39, 40, 42). Thirdly, the use of different anti-B7-H3 antibodies and immunohistochemistry protocols might account for differences in reported expression rates. We used antibody clone SP206, which has been generated by immunization with a synthetic peptide derived from the C-terminus of B7-H3, which is present in both B7-H3 isoforms, 2Ig-B7-H3 and 4Ig-B7-H3. Therefore, this antibody recognizes both isoforms. The specificity of this antibody has been established previously in two independent studies (33, 44), and we validated our immunohistochemistry protocol by staining of several healthy tissues. In contrast, most previously published studies on B7-H3 expression in pancreatic cancer do not show any data on validation of their B7-H3 immunohistochemistry protocol (24, 36–38, 40–42), and/or do not even mention the anti-B7-H3 antibody clone used (37, 40, 42). It is therefore difficult to judge the accuracy of the data reported by these studies. Our data reliably show that B7-H3 is infrequently expressed in cancer cells of Caucasian pancreatic carcinoma patients, while expression in tumor stroma is more common.

The conflicting results regarding associations between tumoral B7-H3 expression and prognosis of pancreatic cancer patients reported by previous studies may also be related to the same technical issues and/or differences between patient cohorts. Although our pancreatic cancer patient cohort was much larger (137 patients) than most of the previous cohorts in which association between B7-H3 expression and prognosis was investigated (36, 39–42), we found no association between B7-H3 expression in cancer cells or in tumor stroma with cancer recurrence or patient survival. The only other study that included a patient cohort of similar size (150 patients) (32) showed that B7-H3 expression on tumor cells was associated with worse survival, but the patients were of Asian origin.

Even though pancreatic cancer and ampullary cancer of the pancreato-biliary subtype are histologically almost indistinguishable, we found that B7-H3 is more frequently expressed in cancer cells of ampullary cancer patients compared to pancreatic cancer patients. Similarly, we have previously reported that cancer cell expression of the co-inhibitory immune checkpoint molecules PDL-1 and HHLA-2 is more prevalent in ampullary cancer patients than in pancreatic cancer patients (p=0.043) (50). These data indicate that ampullary tumors differ from pancreatic tumors in terms of higher expression rate of at least three different co-inhibitory molecules of the B7 superfamily in their cancer cells.

While an inverse association between cancer cell expression of a co-inhibitory molecule and patient prognosis is counter-intuitive, such association may be explained by the phenomenon of adaptive immune resistance. This means that tumor cells up-regulate expression of inhibitory immune checkpoint molecules in response to T-cell infiltration in order to evade immune attack (52, 53). Thereby, inhibitory immune checkpoint expression on cancer cells is a sign of anti-tumor immune pressure. Indeed, expression of the well-studied co-inhibitory B7 family member PD-L1 has been found to be associated with better patient outcome in several cancer types, including pancreatic cancer (43, 52, 54). Mechanistically, this phenomenon is caused by induction of PD-L1 expression on tumor cells by cytokines such as IFN-γ that are secreted by tumor-infiltrating T cells (55). Similarly, IFN-γ has been shown to enhance B7-H3 expression on pancreatic cancer cell lines, and B7-H3 mRNA and IFN-γ mRNA levels were found to correlate positively in pancreatic cancer (36).

The majority of pancreatic tumors and ampullary tumors show prominent B7-H3 expression on tumor stroma. A previous study revealed that B7-H3 in pancreatic cancer is expressed by tumor vasculature (30), while in breast cancer and ovarian cancer B7-H3 was shown to be expressed by tumor-associated fibroblasts (33, 56). It is beyond the scope of the present study to investigate whether in ampullary cancer B7-H3 is expressed in endothelial cells, fibroblasts, or both. Nevertheless, the possibility of eradicating tumor stroma by targeting B7-H3 can be advantageous, as tumor stroma can promote tumor growth (34, 35).

In conclusion, B7-H3 may represent an interesting potential immunotherapeutic target for ampullary cancer of the pancreato-biliary subtype, because it is expressed in cancer cells of about half of the patients, and in about two-thirds of patients in tumoral stroma. In contrast, in pancreatic cancer patients, B7-H3 is less frequently expressed in cancer cells and its expression in tumor stroma is lower. Together, these data are in favor of exploiting therapeutic targeting of B7-H3 in ampullary cancer rather than in pancreatic cancer.

## Data Availability Statement

The raw data supporting the conclusions of this article will be made available by the authors, without undue reservation.

## Ethics Statement

The studies involving human participants were reviewed and approved by METC Erasmus Medical Center Rotterdam. The patients/participants provided their written informed consent to participate in this study.

## Author Contributions

KS, DS, MB, PB, and JK contributed to the design and conduct. EG, KS, MA, and PB contributed to the acquisition of the data. EG, MA, KS, and PB contributed to the analysis. CV contributed to material support. PB and JK supervised the study. All authors contributed substantial to the interpretation of data and were involved in drafting and revising the manuscript. All authors contributed to the article and approved the submitted version.

## Funding

The study was funded by Erasmus MC, Rotterdam.

## Conflict of Interest

The authors declare that the research was conducted in the absence of any commercial or financial relationships that could be construed as a potential conflict of interest.
